# Epitope-Based Peptide Vaccine against Glycoprotein G of Nipah *Henipavirus* Using Immunoinformatics Approaches

**DOI:** 10.1155/2020/2567957

**Published:** 2020-04-22

**Authors:** Arwa A. Mohammed, Shaza W. Shantier, Mujahed I. Mustafa, Hind K. Osman, Hashim E. Elmansi, Isam-Aldin A. Osman, Rawan A. Mohammed, Fatima A. Abdelrhman, Mihad E. Elnnewery, Einas M. Yousif, Marwa M. Mustafa, Nafisa M. Elfadol, Alaa I. Abdalla, Eiman Mahmoud, Ahmed A. Yagaub, Yassir A. Ahmed, Mohamed A. Hassan

**Affiliations:** ^1^Department of Biotechnology, Africa City of Technology, Sudan; ^2^Department of Pharmacy, Sudan Medical Council, Khartoum, Sudan; ^3^Department of Pharmaceutical Chemistry, Faculty of Pharmacy, University of Khartoum, Sudan; ^4^Department of Pharmaceutics, Faculty of Pharmacy, University of Khartoum, Sudan; ^5^Department of Molecular Biology, Institute of Endemic Diseases, University of Khartoum, Sudan; ^6^Department of Bioinformatics, Faculty of Information and Science Technology, Multimedia University, Malaysia; ^7^Department of Biochemistry, Faculty of Science, Bahri University, Sudan; ^8^Department of Clinical Chemistry, Faculty of Medical Laboratory Sciences, Omdurman Islamic University, Sudan; ^9^Department of Microbiology, Faculty of Medical Laboratory Sciences, Omdurman Islamic University, Sudan; ^10^Department of Immunology, Faculty of Medicine, Ahfad University for Women, Sudan; ^11^Department of Bioinformatics, DETAGEN Genetics Diagnostic Center, Kayseri, Turkey

## Abstract

**Background:**

Nipah belongs to the genus *Henipavirus* and the *Paramyxoviridae family*. It is an endemic most commonly found at South Asia and has first emerged in Malaysia in 1998. Bats are found to be the main reservoir for this virus, causing disease in both humans and animals. The last outbreak has occurred in May 2018 in Kerala. It is characterized by high pathogenicity and fatality rates which varies from 40% to 70% depending on the severity of the disease and on the availability of adequate healthcare facilities. Currently, there are no antiviral drugs available for NiV disease and the treatment is just supportive. Clinical presentations for this virus range from asymptomatic infection to fatal encephalitis.

**Objective:**

This study is aimed at predicting an effective epitope-based vaccine against glycoprotein G of Nipah henipavirus, using immunoinformatics approaches.

**Methods and Materials:**

Glycoprotein G of the Nipah virus sequence was retrieved from NCBI. Different prediction tools were used to analyze the epitopes, namely, BepiPred-2.0: Sequential B Cell Epitope Predictor for B cell and T cell MHC classes II and I. Then, the proposed peptides were docked using Autodock 4.0 software program. *Results and Conclusions*. The two peptides TVYHCSAVY and FLIDRINWI have showed a very strong binding affinity to MHC class I and MHC class II alleles. Furthermore, considering the conservancy, the affinity, and the population coverage, the peptide FLIDRINWIT is highly suitable to be utilized to formulate a new vaccine against glycoprotein G of Nipah henipavirus. An in vivo study for the proposed peptides is also highly recommended.

## 1. Introduction

Nipah virus (NiV) is an RNA virus that belongs to the genus *Henipavirus* within the family *Paramyxoviridae* and has first emerged in Malaysia in 1998, gaining its name from a village called Sungai Nipah where it was isolated from the cerebrospinal fluid (CSF) of one of the patients [1–4]. NiV is transmitted zoonotically (from bats to humans, or from bats to pigs, and then to humans) as well as human-to-human routes. Its clinical presentation varies from asymptomatic (subclinical) infection to acute respiratory illnesses and fatal encephalitis in most of the patients who has been in direct contact with infected pigs. It has also been found that the virus causes central nervous system illnesses in pigs and respiratory illnesses in horses resulting in a significant economic loss for farmers [1, 5–9]. Large fruit bats of the genus *Pteropus* seem to act as a natural reservoir of NiV based on the isolation of Hendra virus which has showed the presence of neutralizing antibodies to the Hendra virus on the bats [10, 11]. Although, there are no more cases of NiV in Malaysia, several outbreaks have been frequently occurring in India, Bangladesh, Thailand, and Cambodia [12]. The case fatality rate ranges from 50% to 100%, making it one of the deadliest viruses known to infect humans [3, 13, 14].

Laboratory diagnosis of Nipah virus infection is made using reverse transcriptase polymerase chain reaction (RT-PCR) from throat swabs, cerebrospinal fluid, urine, and blood analysis during acute and convalescent stages of the disease. IgG and IgM antibody detection can be done after recovery to confirm Nipah virus infection. Immunohistochemistry on tissues collected during an autopsy can also confirm the disease [15, 16]. Currently, there are no effective treatments for the Nipah virus infection. Therefore, a few precautions should be followed such as practicing standard infection control, barrier nursing to avoid the spread of the infection from person to person, and the isolation of those suspected to have the infection [7, 8, 17]. Recent computational approaches have provided further information about viruses, including the study conducted by Badawi M et al. on Zika virus, where the envelope glycoprotein was obtained using protein databases. The most immunogenic epitope for the T and B cells involved in cell-mediated immunity was previously analyzed [18]. The main focus of the analysis was the MHC class I potential peptides using in silico analysis techniques [19, 20]. In this study, the same techniques were applied to keep MHC classes I and II along with the world population coverage as our main focus. Furthermore, in this study, we aimed to design an epitope-based peptide vaccine against Nipah virus using peptides of its glycoprotein G as an immunogenic part to stimulate a protective immune response [3].

Nipah virus invades host cells by the fusion of the host cell membranes at an optimal physiological pH for cleavage without requiring viral endocytosis. Cell-cell fusion is a pathological lineament of Nipah virus infections, resulting in a cell-to-cell spread, inflammation, and destruction of endothelial cells and neurons [21]. Both Nipah virus entry and cell-cell fusion require concerted efforts of the attachment of glycoprotein G and fusion (F) glycoprotein. Upon receptor binding, Nipah virus glycoprotein G triggers a conformational cascade in Nipah virus glycoprotein F that executes a viral and/or a cell membrane fusion [22]. Due to the potency of glycoprotein G over F, we have considered this incident to be the target of this study. There are a lot of challenges regarding the development of peptide-based vaccines, and therefore, we have decided to study and propose a new vaccine against the Nipah virus, since they make a helpful alternative strategy that relies on the usage of short peptide fragments to induce immune responses [23–26]. Antigenic epitopes from single proteins may not be really necessary, whereas some of these epitopes may even be detrimental to the induction of protective immunity. This logic has created an interest in peptide vaccines and especially those containing only epitopes that are capable of inducing desirable T cell- and B cell-mediated immune responses. Less than 20 amino acid sequences make up the peptides used in such vaccines, which are then synthesized to form an immunogenic peptide molecule. These molecules represent a specific epitope of an antigen. These vaccines are also capable of inducing immunity against different strains of a specific pathogen by forming noncontiguous and immunodominant epitopes that are usually conserved in the strains of the pathogen [27].

The production of peptide vaccines is extremely safe and cost-effective, especially when they are compared to conventional vaccines. Traditional vaccines that prevent emerging infectious diseases (EIDs) are very difficult to produce because they require the need to culture pathogenic viruses in vitro. However, epitope-based peptide vaccines do not require any means of in vitro culturing which makes them biologically safe, allowing a large scale of bioprocessing to be carried out rapidly and economically. Finally, their selectivity allows a precise activation of the immunological responses by means of selecting immunodominant and conserved epitopes [25, 28]. The complexity of an epitope-based peptide vaccines' design depends largely on the properties of its carrier molecules' reactogenicity as well as its allergenicity [29, 30]. When it comes to the selection of epitopes, it is based on the analysis of the B cells, cytotoxic T cells, and the induction of the helper T cells. Then, it is important to identify the epitopes capable of activating T cells vital for stimulating a protective immunity. One of the issues concerning peptide vaccines representing T cells in a human population and that are highly MHC-heterogeneous is to identify the highly conserved immunodominant epitopes that are considered to be among a broad spectrum of vaccines due to their ability to work against multiple serovars of pathogens [30]. In this study, we have used a variety of bioinformatics tools for the prediction of epitopes along with the population coverage and epitope selection algorithms, including the translocation of peptides into MHC class I and MHC class II.

## 2. Materials and Methods

### 2.1. Sequence Retrieval

The amino acid sequences of glycoprotein G (Glycoside hydrolase family) for a total of 21 strains of Nipah virus were retrieved from the NCBI database (https://www.ncbi.nlm.nih.gov/protein) [31] in a FASTA format on July 2018. Different prediction tools of Immune Epitope Database (IEDB) Analysis Resource (http://www.iedb.org/) [32] were then used to analyze the candidate epitopes.

### 2.2. Conservation Region and Physicochemical Properties

Conservation regions were determined using multiple sequence alignments with the help of Clustal-W in the BioEdit software version 7.2.5 [33]. Epitope conservancy prediction for individual epitopes was then calculated using the IEDB Analysis Resource. Conservancy can be defined as the portion of a protein sequence that restrains in which an epitope is measured at or which that is exceeding a specific level of identity [34]. The physicochemical properties of the retrieved sequence, molecular weight, and amino acid composition were also determined by using BioEdit software.

### 2.3. B Cell Epitope Prediction Tools

Candidate epitopes were analyzed using several B cell prediction methods to determine their antigenicity, flexibility, hydrophilicity, and surface accessibility. The predicted linear epitopes were obtained from the Immune Epitope Database (http://tools.iedb.org/bcell/result/) [35] using a BepiPred test with a threshold value of 0.149 and a window size of 6.0. Moreover, surface accessible epitopes were predicated with a threshold value of 1.0 and a window size of 6.0 using the Emini surface accessibility prediction tool [35]. Kolaskar and Tongaonkar antigenicity methods (http://tools.iedb.org/bcell/result/) were also proposed to determine the sites of antigenic epitopes with a default threshold value of 1.030 and a window size 6.0 [36].

### 2.4. T Cell Epitope Prediction Tools

#### 2.4.1. Peptide Binding to MHC Class I Molecules

The binding peptide was assessed by the IEDB MHC I prediction tool at http://tools.iedb.org/mhcI. This tool employs different methods to determine the ability of the submitted sequence to bind to a specific MHC class I molecule. The artificial neural network (ANN) method was used to calculate IC50 values of the peptide binding to MHC class I molecules. For both frequent and nonfrequent alleles, the peptide length was set to 9 amino acids prior to the prediction. The alleles having a binding affinity of IC50 that are equal to or less than 500 nM were considered for further analysis [37].

#### 2.4.2. Peptide Binding to MHC Class II Molecules

To predict the peptide binding to MHC class II molecules, the MHC II prediction tool http://tools.iedb.org/mhcII provided by the Immune Epitope Database (IEDB) Analysis Resource consisting of human allele references sets was used [38]. The artificial neural network prediction method was chosen to identify the binding affinity of MHC II grooves and MHC II binding core epitopes. All epitopes that bind to many alleles at a score equal to or less than 1000, half-maximal inhibitory concentration (IC50), were selected for further analysis.

### 2.5. Population Coverage

The population coverage of each epitope was calculated by the IEDB population coverage tool at (http://tools.iedb.org/tools/population/iedb_input). This tool was used in order to determine the fraction of individuals predicted to respond to a given set of epitopes, with known MHC restrictions [39]. For every single population coverage, the tool computed the following information: (1) predicted population coverage, (2) HLA combinations recognized by the population, and (3) HLA combinations recognized by 90% of the population (PC90). All the epitopes and their MHC I and MHC II molecules were assessed against the population coverage area selected before submission.

### 2.6. Homology Modeling

The 3D structure of glycoprotein G of Nipah virus was predicted using the RaptorX web portal (http://raptorx.uchicago.edu/), where the reference sequence was submitted in a FASTA format on 14/9/2018 and the structure was received on 15/9/2018 [40]. This structure was then treated with UCSF Chimera 1.10.2 to visualize the position of the proposed peptides [41].

### 2.7. In Silico Molecular Docking

#### 2.7.1. Ligand Preparation

In order to estimate the binding affinities between the epitopes and molecular structures of MHC I and MHC II, we have carried out an in silico molecular docking. Sequences of proposed epitopes were then selected from the Nipah virus reference sequence using Chimera 1.10 and saved as a (pdb) file. The obtained files were then optimized and energy minimized. The HLA-A^∗^02:01 was selected as the macromolecule for docking. Its crystal structure (4UQ3) was downloaded from the RCSB Protein Data Bank (http://www.rcsb.org/pdb/home/home.do), which was in a complex with an azobenzene-containing peptide [42].

All water molecules and heteroatoms in the retrieved target file 4UQ3 were then removed. The target structure was further optimized and energy minimized using Swiss PDB viewer V.4.1.0 software [43].

Molecular docking was performed using AutoDock 4.0 software, based on the Lamarckian genetic algorithm, which combines energy evaluation through grids of affinity potential to find the suitable binding position for a ligand on a given protein [44, 45]. Polar hydrogen atoms were added to the protein targets, and Kollman united atomic charges were computed. The targets' grid map was calculated and set to 60 × 60 × 60 points with a grid spacing of 0.375 Ǻ. The grid box was then allocated properly in the target to include the active residue in the center. The genetic algorithm and its run were set to 100 as the docking algorithms were set on default. Finally, results were retrieved as binding energies and poses that showed the lowest binding energies in which they were visualized using UCSF Chimera.

## 3. Results

### 3.1. Nipah Virus Glycoprotein G Physical and Chemical Parameters

The physicochemical properties of the Nipah virus glycoprotein G protein was assessed using BioEdit software version 7.0.9.0. The protein length was found to be 602 amino acids, and the molecular weight was at 67035.54 Daltons. The amino acids that form the Nipah virus glycoprotein G protein are shown in Table 1 along with their numbers and molar percentages in (Mol%).

### 3.2. B Cell Epitope Prediction

**Figure 1:**
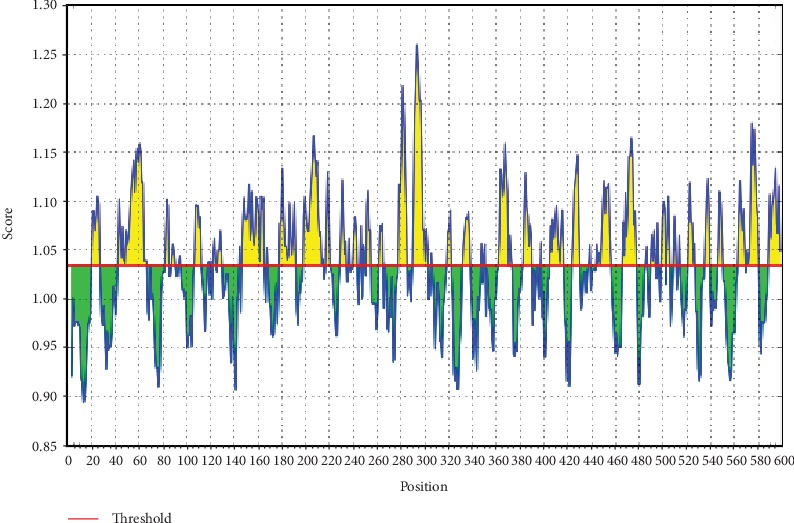
BepiPred linear prediction. Areas above the red line (threshold) are epitopes suggested to be binding to the B cells while the green areas are not.

**Figure 2:**
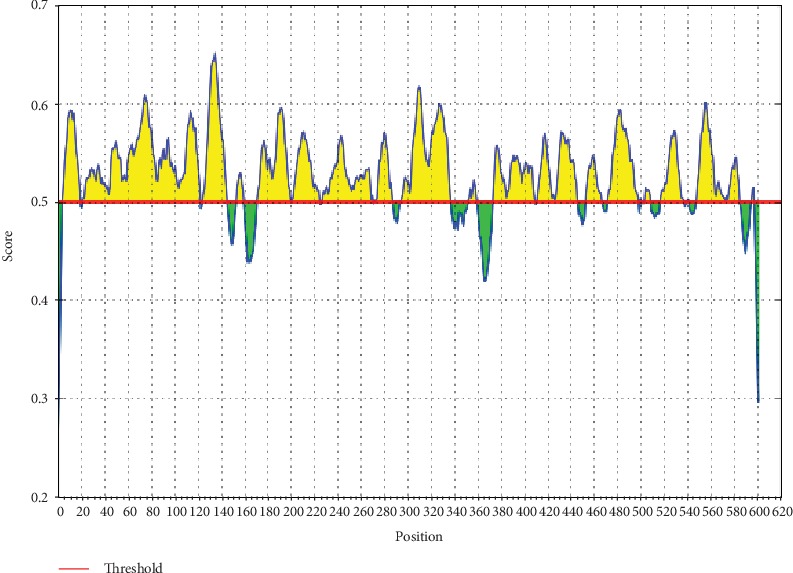
Emini surface accessibility prediction. Areas above the red line (threshold) are epitopes suggested to be binding to the B cells while the green areas are not.

**Figure 3:**
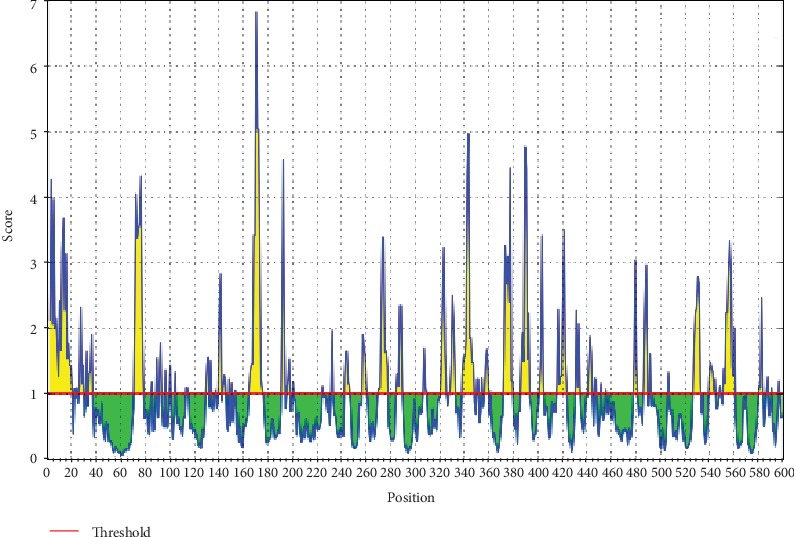
Kolaskar and Tongaonkar antigenicity prediction. Areas above the red line (threshold) are epitopes suggested to be binding to the B cells while the green areas are not.

The ref sequence of the Nipah virus glycoprotein G was subjected to a Bepipred linear epitope prediction. Emini surface accessibility and Kolaskar and Tongaonkar antigenicity methods in IEDB were used to determine bindings to the B cell and in testing its surface and immunogenicity. The results are shown in Figures 1–3.

### 3.3. Prediction of T Helper Cell Epitopes and Interaction with MHC Class I Alleles

The Nipah virus glycoprotein G sequence was analyzed using the IEDB MHC class I binding prediction tool based on ANN-align with half-maximal inhibitory concentration (IC_50_) ≤ 500; the least most promising epitopes that had a binding affinity with the class I alleles along with their positions in the Nipah virus glycoprotein G are shown in Table 2.

### 3.4. Prediction of T Helper Cell Epitopes and Interaction with MHC Class II Alleles

The Nipah virus glycoprotein G sequence was analyzed using the IEDB MHC class II binding prediction tool based on NN-align with half-maximal inhibitory concentration (IC_50_) ≤ 1000. The list of the epitopes and their correspondent bindings to MHC class II alleles, along with their positions in the Nipah virus glycoprotein G, while the list of the most promising epitopes that had a strong binding affinity to MHC class II alleles and depending on the number of their binding alleles is shown in Table 3.

### 3.5. Population Coverage

A population coverage test was performed to detect all the epitopes that bind to MHC class I alleles and MHC class II alleles available in the database in relation to the world, South Asia, Southeast Asia, Sudan, and North Africa.

### 3.6. 3D Structure

### 3.7. Molecular Docking

## 4. Discussion

Traditional vaccination approaches depend on the total amount of pathogens that are either live—constricted or inactivated. Among the significant issues, these vaccines have brought along pivotal security concerns. In light of the fact that they are being utilized for vaccination, this may have caused them to become actuated and may also cause contamination. Additionally, due to the varied hereditary pathogen strains found in the world, vaccines are probably going to lose their viability in various areas or even in certain populations.

However, novel vaccine approaches such as DNA- and epitope-based immunizations may possibly conquer obstructions for this type of immunization approaches, making them increasingly successful, explicit, and long-lasting in vulnerable reactions with insignificant structures and without any undesired impacts [46]. Moreover, many peptide-based vaccines have been effectively proposed through utilizing in silico approaches against *Madurella mycetomatis*, Mokola rabies virus, Lagos rabies virus, and others [47–52]. Such investigations, in regard to those viruses, have built up immunoinformatics in the computational analysis field.

**Figure 4:**
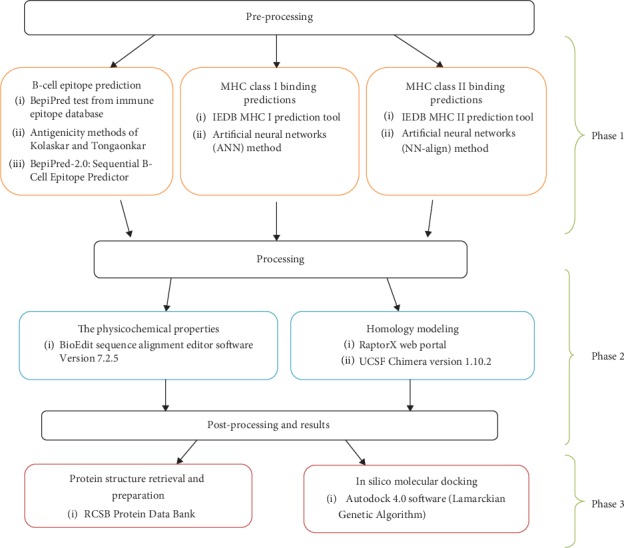
The three phases of Materials and Methods.

In our present work, potential peptides were suggested to design an epitope-based vaccine for Nipah virus, using the latest amino acid sequences of glycoprotein G (glycoside hydrolase family) for a total of 21 strains of Nipah virus that were retrieved from the NCBI database (https://www.ncbi.nlm.nih.gov/protein) [31] on July 2018 after the last outbreak at the end of May 2018 in Kerala-India according to the WHO report [53].  Figure 4 summarizes the method of the present work.

Various literatures were surveyed to define the antigenic part of the virus. Glycoprotein G was found to be on the outer surface of the virus which was chosen as our target. Initially, we have evaluated the binding affinity of the virus to MHC alleles. This was done by submitting the protein reference sequence to IEDB MHC, a binding prediction tool, based on the ANN align method with IC_50_ ≤ 500 [37] for MHC class I molecules. 191 peptides were found to bind to MHC class I with different affinities. It is well known that a better immune response depends on whether or not the recognition of epitopes by HLA molecules with significant affinity is successful. Therefore, a peptide recognized by its highest number of HLA alleles has the best potential to induce a strong immune response, leading us to take into account the only three peptides found with a 100% conservancy. The conserved peptide FLIDRINWI was found to interact with 8 alleles (HLA-A^∗^02:01, HLA-A^∗^02:03, HLA-A^∗^02:06, HLA-A^∗^68:02, HLA-C^∗^03:03, HLA-C^∗^06:02, HLA-C^∗^07:01, and HLA-C^∗^12:03), while FSWDTMIKF with 8 alleles (HLA-A^∗^02:06, HLA-A^∗^29:02, HLA-B^∗^35:01, HLA-B^∗^46:01, HLA-B^∗^53:01, HLA-B^∗^57:01, HLA-B^∗^58:01, and HLA-C^∗^12:03) and TVYHCSAVY with 11 alleles (HLA-A^∗^03:01, HLA-A^∗^11:01, HLA-A^∗^26:01, HLA-A^∗^29:02, HLA-A^∗^30:02, HLA-A^∗^68:01, HLA-B^∗^15:01, HLA-B^∗^15:02, HLA-B^∗^35:01, HLA-C^∗^12:03, and HLA-C^∗^14:02).

The reference sequence of Nipah virus glycoprotein G was reanalyzed using the IEDB MHC II binding prediction tool based on NN-align with half-maximal inhibitory concentration (IC50) ≤ 1000 [38]. The analysis resulted in the prediction of 398 peptides from which FSWDTMIKF, FLIDRINWI, and ILSAFNTVI were potentially proposed according to their high number of binding alleles (15, 12, and 15 alleles, respectively). Additionally, the sequence of Nipah virus glycoprotein G was subjected to BepiPred linear epitope prediction, Emini surface accessibility, and Kolaskar and Tongaonkar antigenicity methods in IEDB. Unfortunately, the peptides with the strongest binding affinities, utilizing the three mentioned tests, were absent.

Population coverage results for the total peptides found and the proposed peptides binding to MHC classes I and II alleles are summarized in Tables 4 and 5. Obtained results from the bindings to MHC I alleles revealed a 99.84% projected population coverage in the world, 98.55% in Southeast Asia, 98.40% in South Asia, 99.23% in North Africa, and 99.36% in Sudan while the population coverage results for the total number of peptides binding to MHC II alleles showed only a 56.84% projected population coverage in the world, 48.63% in Southeast Asia, 56.00% in South Asia, 62.37% in North Africa, and 55.75% in Sudan.

The selected peptides were further subjected to both MHC I- and MHC II-based population coverage analysis in the whole world, Southeast Asia, South Asia, North Africa, and Sudan as shown in Table 5. Among the six primarily selected epitopes, the obtained results showed a very strong potential in proposing the epitope FLIDRINWI as a vaccine candidate compared to the rest, taking into consideration its overall epitope conservancy, population coverage, and its affinity for the highest number of HLA molecules. Furthermore, in silico docking was carried out to measure the binding efficacy between the proposed peptides and HLA-A^∗^02:01, in which it has been specifically chosen in relation to their contribution to several immunological and pathological diseases [54–56], although numerous investigations have shown a relationship between HLA alleles and disease susceptibility, which defines defensive HLA allelic associations that possibly permit a recognizable proof that pathogen epitopes are limited by particular HLA alleles. These epitopes may then be fused into a vaccine design in the expectation that the immunization will be reproduced naturally [55, 56].

**Figure 5:**
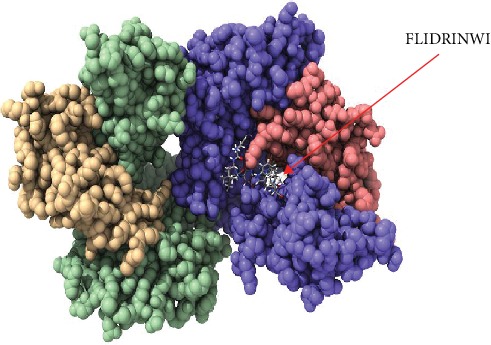
Molecular docking of FLIDRINWI peptide of Nipah virus docked in HLA-A^∗^02:01 and visualized by UCSF Chimera X version 0.1.0.

Calculations of the root mean square deviation (RMSD) between coordinates of the atoms and formation of clusters based on RMSD values have computed the resemblance of the docked structures. The most favorable docking is considered to be the conformation of the lowest binding energy. The least energy predictions of the peptide FLIDRINWI (-6.95 Kcal/mol) and the 3D structure of the allele and its peptide are shown in Figure 5. Furthermore, the monoisotopic mass, sum formula, and molecular weight of the three highly proposed peptides are shown in Table 6.

As a result of these interesting outcomes, formulating a vaccine using the suggested peptide is highly promising and encouraging to be highly proposed as a universal epitope-based peptide vaccine against Nipah virus.

## 5. Conclusions

The present study proposed a very promising epitope-based peptide vaccine against glycoprotein G of Nipah virus. It is expected to be highly antigenic with a minimum allergic effect. The proposed peptide FLIDRINWI has a strong binding affinity to both MHC class I and MHC class II alleles. Moreover, it shows an exceptional population coverage result for both MHC class I and MHC class II alleles in the whole world, Southeast Asia, South Asia, North Africa, and Sudan.

**Figure 6:**
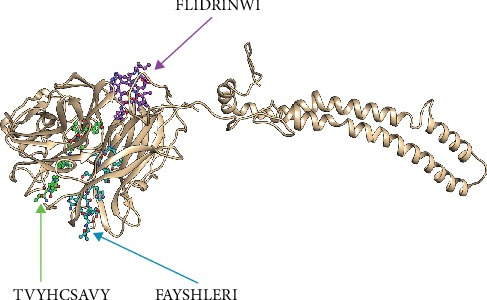
The four potential peptides bound to MHC class I and MHC class II visualized by Chimera X version 0.1.0.

**Figure 7:**
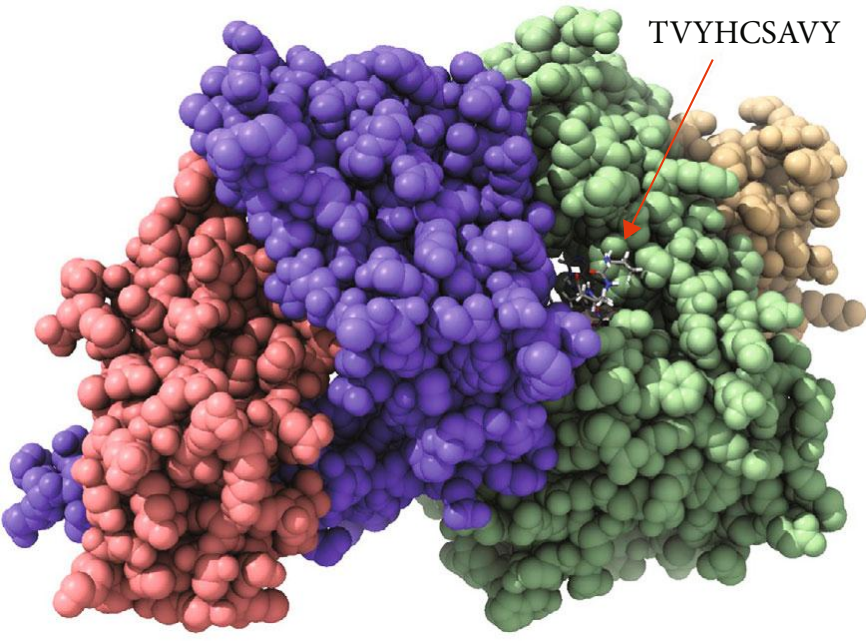
Molecular docking of TVYHCSAVY peptide of Nipah virus docked in HLA-A^∗^02:01 and visualized by UCSF Chimera X version 0.1.0.

**Figure 8:**
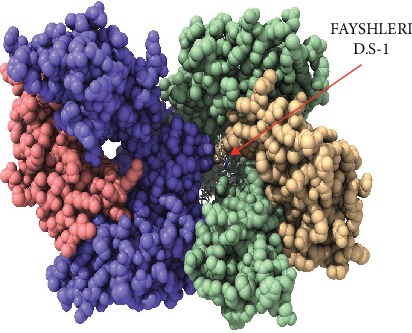
Molecular docking of FAYSHLERI peptide of Nipah virus docked in HLA-A^∗^02:01 and visualized by UCSF Chimera X version 0.1.0. ^∗^D.S: Docking Side No.1.

**Figure 9:**
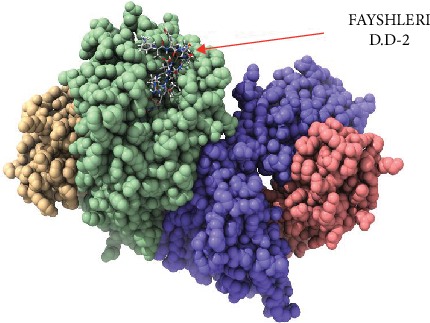
Molecular docking of FAYSHLERI peptide of Nipah virus docked in HLA-A^∗^02:01 and visualized by UCSF Chimera X version 0.1.0. ^∗^D.S: Docking Side No.2.

Despite having to validate the findings of the current study, an in vivo assessment of the most promising peptides, namely, FLIDRINWI, TVYHCSAVY, and FAYSHLERI, is highly recommended and will serve as the ground data for such work as shown in Figures 5–9.

## Figures and Tables

**Table 1:** Number and Mol% of amino acids that constituted *Nipah virus glycoprotein G* using BioEdit software version 7.2.5.

Amino acid	Number	Mol%	Amino acid	Number	Mol%
Ala A	23	3.82	Met M	11	1.83
Cys C	17	2.82	Asn N	45	7.48
Asp D	27	4.49	Pro P	36	5.98
Glu E	26	4.32	Gln Q	25	4.15
Phe F	21	3.49	Arg R	22	3.65
Gly G	40	6.64	Ser S	51	8.47
His H	5	0.83	Thr T	37	6.15
Ile I	55	9.14	Val V	41	6.81
Lys K	39	6.48	Trp W	7	1.16
Leu L	49	8.14	Tyr Y	25	4.15

**Table 2:** Most potential T cell epitopes interacting with MHC class I alleles, their positions, IC50, rank, and conservancy.

Peptide	Start	End	Allele	IC50	Rank	Conservancy
FAYSHLERI	229	237	HLA-A^∗^02:01	376.44	2.5	
	229	237	HLA-A^∗^02:03	19.03	0.37	
	229	237	HLA-A^∗^02:06	23.3	0.28	76.19
	229	237	HLA-A^∗^68:02	104.31	0.63	
	229	237	HLA-B^∗^51:01	133.03	0.02	
	229	237	HLA-B^∗^53:01	222.39	0.21	
	229	237	HLA-C^∗^03:03	57.38	0.22	
	229	237	HLA-C^∗^06:02	112.27	0.05	
	229	237	HLA-C^∗^07:01	151.15	0.05	
	229	237	HLA-C^∗^12:03	13.37	0.03	
	229	237	HLA-C^∗^15:02	207.16	0.12	

FLIDRINWI	512	520	HLA-A^∗^02:01	3.65	0.02	
	512	520	HLA-A^∗^02:03	2.32	0.02	
	512	520	HLA-A^∗^02:06	4.71	0.04	100
	512	520	HLA-A^∗^68:02	488.4	1.8	
	512	520	HLA-C^∗^03:03	366.48	0.63	
	512	520	HLA-C^∗^06:02	163.19	0.07	
	512	520	HLA-C^∗^07:01	416.5	0.12	
	512	520	HLA-C^∗^12:03	55.27	0.13	

FSWDTMIKF	458	466	HLA-A^∗^02:06	131.35	1.3	
	458	466	HLA-A^∗^29:02	328.77	1.2	
	458	466	HLA-B^∗^35:01	70.16	0.24	100
	458	466	HLA-B^∗^46:01	470.72	0.09	
	458	466	HLA-B^∗^53:01	95.8	0.12	
	458	466	HLA-B^∗^57:01	380.38	0.93	
	458	466	HLA-B^∗^58:01	313.77	0.77	
	458	466	HLA-C^∗^12:03	35.09	0.09	

KLISYTLPV	201	209	HLA-A^∗^02:01	2.36	0.02	
	201	209	HLA-A^∗^02:03	2.4	0.02	
	201	209	HLA-A^∗^02:06	3.77	0.02	100
	201	209	HLA-A^∗^30:01	146.34	0.44	
	201	209	HLA-A^∗^32:01	21.39	0.04	
	201	209	HLA-B^∗^15:01	303.74	1.3	
	201	209	HLA-C^∗^14:02	187.05	0.28	
	201	209	HLA-C^∗^15:02	364.98	0.2	

TVYHCSAVY	278	286	HLA-A^∗^03:01	84.04	0.35	
	278	286	HLA-A^∗^11:01	263.53	1.6	
	278	286	HLA-A^∗^26:01	363.08	0.19	
	278	286	HLA-A^∗^29:02	10.13	0.08	100
	278	286	HLA-A^∗^30:02	32.49	0.07	
	278	286	HLA-A^∗^68:01	310.51	1.7	
	278	286	HLA-B^∗^15:01	71.72	0.41	
	278	286	HLA-B^∗^15:02	353.64	0.13	
	278	286	HLA-B^∗^35:01	27.98	0.12	
	278	286	HLA-C^∗^12:03	45.95	0.11	
	278	286	HLA-C^∗^14:02	103.08	0.18	

**Table 3:** The most potential T cell epitopes (core sequence) and the number of their binding alleles.

Core sequence	Alleles	Number of alleles
FLIDRINWI	HLA-DPA1^∗^01/DPB1^∗^04:01	12
	HLA-DPA1^∗^01:03/DPB1^∗^02:01	
	HLA-DPA1^∗^02:01/DPB1^∗^01:01	
	HLA-DPA1^∗^02:01/DPB1^∗^05:01	
	HLA-DPA1^∗^03:01/DPB1^∗^04:02	
	HLA-DQA1^∗^01:01/DQB1^∗^05:01	
	HLA-DQA1^∗^05:01/DQB1^∗^02:01	
	HLA-DRB1^∗^01:01	
	HLA-DRB1^∗^03:01	
	HLA-DRB1^∗^04:01	
	HLA-DRB1^∗^04:04	
	HLA-DRB1^∗^04:05	

FAYSHLERI	HLA-DPA1^∗^01:03/DPB1^∗^02:01	13
	HLA-DPA1^∗^02:01/DPB1^∗^01:01	
	HLA-DPA1^∗^02:01/DPB1^∗^05:01	
	HLA-DPA1^∗^03:01/DPB1^∗^04:02	
	HLA-DQA1^∗^05:01/DQB1^∗^02:01	
	HLA-DQA1^∗^05:01/DQB1^∗^03:01	
	HLA-DRB1^∗^01:01	
	HLA-DRB1^∗^04:04	
	HLA-DRB1^∗^04:05	
	HLA-DRB1^∗^07:01	
	HLA-DRB1^∗^09:01	
	HLA-DRB3^∗^01:01	
	HLA-DRB5^∗^01:01	

FIEISDQRL	HLA-DPA1^∗^01:03/DPB1^∗^02:01	17
	HLA-DPA1^∗^02:01/DPB1^∗^01:01	
	HLA-DPA1^∗^03:01/DPB1^∗^04:02	
	HLA-DQA1^∗^05:01/DQB1^∗^02:01	
	HLA-DRB1^∗^01:01	
	HLA-DRB1^∗^04:05	
	HLA-DRB1^∗^07:01	
	HLA-DRB1^∗^08:02	
	HLA-DRB1^∗^13:02	
	HLA-DRB1^∗^15:01	
	HLA-DRB4^∗^01:01	
	HLA-DRB5^∗^01:01	
	HLA-DRB1^∗^04:01	
	HLA-DRB1^∗^07:01	
	HLA-DRB1^∗^09:01	
	HLA-DRB1^∗^11:01	
	HLA-DRB1^∗^13:02	

ILSAFNTVI	HLA-DPA1^∗^03:01/DPB1^∗^04:02	13
	HLA-DQA1^∗^05:01/DQB1^∗^03:01	
	HLA-DRB1^∗^01:01	
	HLA-DRB1^∗^04:01	
	HLA-DRB1^∗^04:05	
	HLA-DRB1^∗^07:01	
	HLA-DRB1^∗^08:02	
	HLA-DRB1^∗^09:01	
	HLA-DRB1^∗^11:01	
	HLA-DRB1^∗^13:02	
	HLA-DRB1^∗^15:01	
	HLA-DRB4^∗^01:01	
	HLA-DRB5^∗^01:01	

TVYHCSAVY	HLA-DQA1^∗^05:01/DQB1^∗^03:01	4
	HLA-DRB1^∗^07:01	
	HLA-DRB1^∗^13:02	
	HLA-DRB1^∗^15:01	

**Table 4:** A population coverage for all epitopes that bind to MHC classes I and II alleles from different parts of the world.

MHC classes	Population	World	South Asia	Southeast Asia	Sudan	North Africa
Class I	Coverage^a^	99.84%	98.40%	98.55%	99.36%	99.23%
	Average_hit^b^	36.62	30.60	28.42	34.02	32.43
	PC90^c^	16.87	9.29	8.61	13.40	12.95

Class II	Coverage^a^	56.84%	56.0%	48.63%	55.75%	62.37%
	Average_hit^b^	54.88	50.50	36.27	36.89	50.09
	PC90^c^	-24.24	-10.09	1.65	4.60	-3.31

^a^Projected population coverage; ^b^average number of epitope hits/HLA combinations recognized by the population; ^c^minimum number of epitope hits/HLA combinations recognized by 90% of the population.

**Table 5:** Population coverage of the three highly proposed peptides in MHC classes I and II in five different parts of the world.

Peptide	Population coverage %/area														
	World			Southeast Asia			South Asia			North Africa			Sudan		
	MHC I	MHC II	MHC I & II	MHC I	MHC II	MHC I & II	MHC I	MHC II	MHC I & II	MHC I	MHC II	MHC I & II	MHC I	MHC II	MHC I & II
FLIDRINWI	70.4%	43.7%	83.3%	44.5%	19.8%	55.5%	51.4%	29.8%	65.9%	71.0%	33.8%	80.8%	85.4%	30.7%	89.9%
FAYSHLERI	74.9%	40.2%	85.0%	50.6%	31.8%	66.3%	60.9%	40.5%	76.7%	77.5%	35.5%	85.5%	89.2%	19.5%	91.3%
TVYHCSAVY	61.3%	40.1%	76.8%	54.5%	18.4%	62.9%	63.7%	45.0%	80.0%	50.4%	43.5%	72.0%	50.2%	20.9%	60.6%

**Table 6:** Monoisotopic mass, sum formula, and molecular weight of the three highly proposed peptides.

Sequence (N : H/C : OH)	Sum formula	Monoisot. mass	Mol. weight
FLIDRINWI	C_58_H_88_N_14_O_13_	1188.66551	1189.40532
TVYHCSAVY	C_47_H_67_N_11_O_14_S	1041.45896	1042.16518
FAYSHLERI	C_53_H_78_N_14_O_14_	1134.58218	1135.27182

## Data Availability

The data used to support the findings of this study are available from the corresponding author upon request.
